# Impact of chronic benzene poisoning on aberrant mitochondrial DNA methylation: A prospective observational study

**DOI:** 10.3389/fpubh.2023.990051

**Published:** 2023-02-03

**Authors:** Dianpeng Wang, Dafeng Lin, Gangquan Feng, Xiangli Yang, Lidan Deng, Peimao Li, Zhimin Zhang, Wen Zhang, Yan Guo, Yue Wang, Song Fu, Naixing Zhang

**Affiliations:** ^1^Medical Laboratory, Shenzhen Prevention and Treatment Center for Occupational Diseases, Shenzhen, China; ^2^Medical Laboratory College, Hebei North University in China, Zhangjiakou, Hebei, China

**Keywords:** benzene poisoning, mitochondrial DNA, methylation, prospective observational study, white blood cells (WBC), platelet

## Abstract

Benzene is used as an industrial solvent, which may result in chronic benzene poisoning (CBP). Several studies suggested that CBP was associated with mitochondrial epigenetic regulation. This study aimed to explore the potential relation between CBP and mitochondrial DNA (mtDNA) methylation. This prospective observational study enrolled CBP patients admitted to Shenzhen Prevention and Treatment Center for Occupational Diseases hospital and healthy individuals between 2018 and 2021. The white blood cell (WBC), red blood cell (RBC), hemoglobin (HB), and platelet (PLT) counts and mtDNA methylation levels were measured using blood flow cytometry and targeted bisulfite sequencing, respectively. A total of 90 participants were recruited, including 30 cases of CBP (20 females, mean age 43.0 ± 8.0 years) and 60 healthy individuals (42 females, mean age 43.5 ± 11.5 years). This study detected 168 mitochondrial methylation sites >0 in all study subjects. The mtDNA methylation levels in the CBP cases were lower than the healthy individuals [median ± interquartile-range (IQR), 25th percentile, 75th percentile: (1.140 ± 0.570, 0.965, 1.535)% vs. median ± IQR, 25th percentile, 75th percentile: (1.705 ± 0.205,1.240,2.445)%, *P* < 0.05]. Additionally, the spearman correlation analysis showed that the mtDNA methylation levels were positively correlated with the counts of circulating leukocytes [WBC (*r* = 0.048, *P* = 0.036)] and platelets [PLT (*r* = 0.129, *P* < 0.01)]. We provided solid evidence of association between CBP and aberrant mtDNA methylation.

## Introduction

Exposure to benzene, an industrial solvent, may inflict chronic benzene poisoning (CBP) on the workers, especially in developing countries, as well as certain lung diseases due to severe hazardous occupational exposure through the respiratory tract (1). Benzene was verified to cause several pathological conditions in humans *via* altering DNA, and it has been classified as a human hematological carcinogen by the International Agency for Research on Cancer (IARC) (2). Metabolism of benzene to toxic metabolites is generally thought to be a critical event in benzene-induced toxicity (3). Benzene-induced hematotoxicity and leukemia may be attributed to these metabolites, which could cause cell apoptosis, mitochondrial dysfunction, and oxidative stress. Benzene could induce DNA alterations in exposed workers including DNA methylation, structural damage such as single and double-strand DNA breaks, and accumulation of point mutations (4, 5). In a previous report, a significantly elevated level of mitochondrial DNA (mtDNA) copy number was observed in the benzene-exposed group compared with in the healthy individuals (6). The changing in mtDNA may be an early molecular event in human cells after they are stimulated by endogenous or exogenous oxidative stress (7). Rapid depolarization of mitochondrial inner membrane potential and subsequent damage to oxidative phosphorylation could be induced by oxidative stress (8). Adults exposed to low levels of benzene have impaired hematopoiesis function (9).

DNA methylation plays a very crucial role in the pathogenesis and progression of CBP (10). Previous research has shown that long-term occupational exposure to benzene with concentration below the allowable exposure limit may still alter DNA methylation of anti-oncogene, which may ultimately lead to the development of cancer (11). A number of biochemical changes are induced by benzene exposure, including changes in histone acetylation and DNA methylation (12). Previously, some studies reported that altered global DNA methylation and endoreduplication were potential mechanisms of aneuploidy induction by the benzene metabolite hydroquinone (HQ) in TK6 cells (13). Signal net analysis of differential methylation genes associated with CBP showed that two key hypomethylated STAT3 was identified (14). The methylation level at the third CpG site of p15 promoter region that may permit unlimited proliferation of the cell and is thought to be associated with leukemogenesis, was significantly higher in the CBP group than in the control group (15). In patients who had occupational CBP, mitochondrial cytochrome *c* oxidase subunit I gene methylation was low and correlated with WBC counts (16). The role of epigenetic regulation of methylation in the pathological regulation of CBP deserves further study. Therefore, based on mitochondrial oxidation, the author hypothesized that aberrant mtDNA methylation might play an important regulatory role in those at risk of exposure to benzene or benzene-related compounds. This study aimed to explore the potential relations between mtDNA methylation and CBP.

## Methods

### Study design and population

This prospective observational study enrolled CBP patients admitted to Shenzhen Prevention and Treatment Center for Occupational Diseases and healthy individuals from several major factories between 2018 and 2021. Written informed consent was obtained from all patients and healthy individuals before sample and data collection. The principle of the Helsinki Declaration for using human subjects was employed. The study protocol was approved by the ethics committee of the hospital.

CBP patients were diagnosed between 2018 and 2021 by the authorized local Occupational Disease Diagnostic Team, with (a) total white blood cell (WBC) counts < 4.0 ± 10^9^/L or WBC counts between 4.0 ± 10^9^/L and 4.5 ± 10^9^/L and platelet (PLT) counts < 80 ± 10^9^/L, and repeated confirmation of these counts in a peripheral blood sample after a few months; (b) documented benzene exposure as a result of employment in a factory for at least 6 months, (c) exclusion of other known causes of abnormal blood cells counts, such as chloromycetin use and ionizing radiation. The diagnostic criteria for occupational CBP are issued by the Ministry of Health and Family Planning Committee (17). Eligible healthy individuals had normal physical examination results and no history of genetic diseases.

### Data collection

The medical records of these patients were independently reviewed by at least two hematopathologists. Each one of the participants donated 2 ml of venous blood and their demographic data were recorded. All subjects underwent a rigorous physical examination.

#### WBC and PLT and HB and RBC determinations

The WBC, PLT, hemoglobin (HB), and red blood cells (RBC) counts were evaluated with SYSMEX automatic blood analyzer (Sysmex Corporation, Kobe, Japan).

#### DNA extraction

Nucleic acids were extracted from the blood samples by extraction DNA Mini kit (Sangon Biotech, Shanghai, China), according to the manufacturer's instruction. DNA quantity was assessed by the spectrometer (Thermo Scientific NanoDrop, NanoDrop Technologies, Wilmington, DE, USA). The qualified DNA was stored at −80°C until analysis.

#### DNA methylation profiling

Genomic DNA was treated for bisulfite sequencing with EpiTect Bisulfite Sequencing kit (Qiagen). Based on the Acegen Targeted Methyl Panel multiple targeted sulfite sequencing system software, and the target sequence (mitochondrial gene ID: NC_012920.1) multiple sulfite PCR amplification primer pool (upstream primer pool A and downstream primer pool B, primer length 26–40 bases, annealing temperature 55–65; the length of the amplicon is 150–250 bp), the sequencing library was prepared using the Acegen Targeted Methyl Panel Kit amplification system. The primer sequences are listed in Supplementary Table S1. First, 50–200 ng of genomic DNA was recovered by sulfite conversion (ZYMO EZ DNA Methylation-Gold Kit, Zymo Research, Irvine, CA, USA) for multiple sulfite PCR amplification for 25–33 cycles; targeted amplification: the product was purified and recovered with 1X magnetic beads (Agencourt AMPure XP, Beckman Coulter) and used for the preparation of small DNA fragment libraries. Acegen DNA Library Prep Kit (Acegen, Cat. No. AG0810) was used for double-end 8 bp-index library construction. The structure is shown in the figure below. The prepared library was subjected to qualitative detection and concentration determination by Qubit 3.0 and Agilent 2100 Bioanalyzer. The qualified library was used for NGS sequencing using the Illumina sequencing system. The sequencing strategy was paired-end index sequencing, and the sequencing read length was PE150; not < 200 ×.

#### Bioinformatics analysis

Qualified data is the basis of information analysis, so quality control of obtained raw data is an essential aspect of data analysis, which mainly includes: (1) Output of raw reads after disembarkation. (2) A clean read is obtained by removing sequencing adapters and low-quality bases and reads using Trimmomatic-0.36. Subsequently, the clean reads and target sequences were aligned and analyzed using methylation sequencing data alignment software (bsmap, version v2.73). The overall statistics are as follows: Raw Q20 (%) average efficiency is 96.45%, clean Q20 (%) average efficiency is 97.62%, mapping_rate (%) average efficiency is 96.45%. All data are >95%.

The methylation level of CG bases on each target sequence of each sample was calculated. The methylation level was calculated according to the following formula: methylation level of C site = number of reads supporting methylation/(supporting methylation number of reads basement + number of reads supporting unmethylation). Detectable site methylation ratio levels for each sample were counted; if the depth was < 30 ×, it would be expressed as NA. And the methylation level of each site in each sample was counted; if the depth was < 30 ×, it would be expressed as NA. Enrichment of functions and signaling pathways of genetic loci with differential methylation expression was performed based on KOBAS v3.0 (http://bioinfo.org/kobas/) using the KEGG database with corrected *P*-value < 0.05.

### Statistical analysis

All analyses were performed with SPSS software version 16.0 (SPSS Inc., Chicago, III., USA). Demographic data, such as age at sampling, and sex, were compared between groups using Student's *t*-test or Fisher's exact test. Mann Whitney's *U*-test were utilized to identify a discrepancy of methylated mtDNA levels between the groups. The correlation between mtDNA methylation levels and blood cells counts was determined using Spearmans correlation coefficient. A two-side *P*-value < 0.05 was considered significant.

## Results

A total of 90 participants were recruited including 30 cases of CBP (20 females, aged 43.0 ± 8.0 years) and 60 healthy individuals (42 females, aged 43.5 ± 11.5 years). There was no difference in age and gender distribution between the CBP patients and healthy individuals (all *P* > 0.05), while the CBP patients had significantly lower blood indices, including WBC count, PLT count, HB concentration, and RBC count (all *P* < 0.001), than the healthy individuals (Table 1).

**Table 1:** Basic profile of the patients and control group.

	**Age (year)**	**Female (%)**	**WBC (10^9^/L)**	**PLT (10^9^/L)**	**HB (g/L)**	**RBC (10^12^/L)**
Cases	43.0 ± 8.0	68.3	3.78 ± 1.387	153.80 ± 58.31	113.33 ± 16.34	3.86 ± 0.65
Controls	43.5 ± 11.5	70	5.74 ± 1.413	244.92 ± 51.99	138.22 ± 13.22	4.89 ± 0.65
*T*	1.565	0.026^^▴^^	6.248	7.524	7.768	7.101
SIG	0.121	0.872	P < 0.001^^*^^	P < 0.001^^*^^	P < 0.001^^*^^	P < 0.001^^*^^

^*^P < 0.05.

^▴^Pearson Chi-square value.

**Figure 1:**
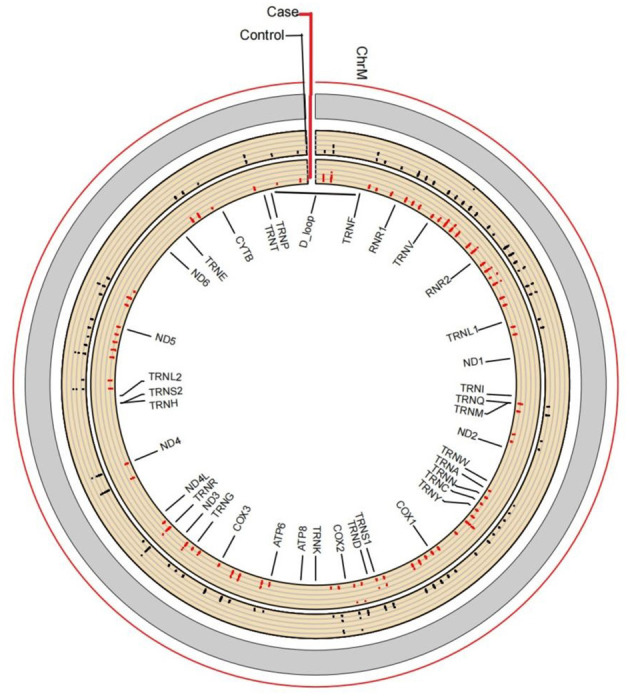
Line chart for visualization of methylation levels. Based on the absolute coordinates of the detected gene region, the methylation level of each locus in each sample is plotted, and marked with different colors according to biological groups, and the average methylation of each locus within the group is calculated. This form more intuitively displays the position of differential methylation on the gene, the average methylation level and the significance of the difference. There are significantly fewer mtDNA methylations in patients with CBP than in healthy individuals in the following regions: RNR1, RNR2, TRNQ, TRNM, TRNW, TRNS1, ND2, NDT, COl, CO2, CO3, ATP6, and CYB.

**Figure 2:**
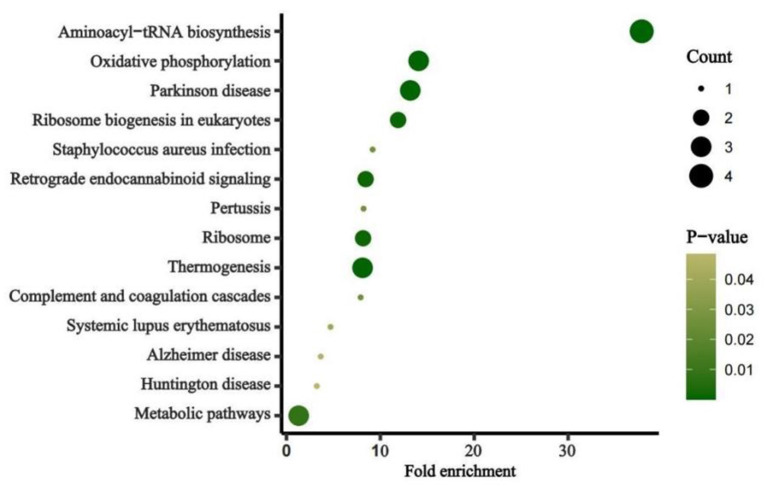
Pathway enrichment of the identified mitochondrion genes. Fold enrichment is calculated by dividing gene ratio (the number of genes enriched in the pathway divided by the total number of analyzed genes) with background ratio in KEGG database. *P*-values were adjusted.

**Figure 3:**
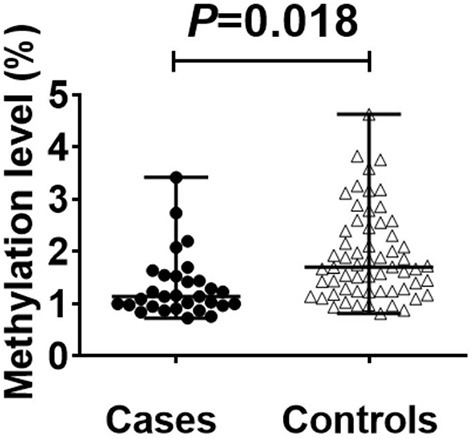
Scatter plot plus bee colony plot showing differences between cases and controls. Statistical analysis by MannWhitney's *U*-test found that there is a significant difference between the cases [(median ± IQR, 25th percentile, 75th percentile: (l.140 ± 0.570, 0.965, 1.535)%] and controls [(median ± IQR, 25th percentile, 75th percentile: (1.705 ± 0.205, l.240, 2.445)] (*P* < 0.05). The methylation site detection results are shown in the figure.

The average read depth of the sequencing data was ≥2,000. The sequencing results showed a total of 168 CpG mtDNA methylation sites >0. The raw sequence can be accessed at https://www.ncbi.nlm.nih.gov/sra/PRJNA877783. The total mtDNA methylation level in the case group was lower than that in the control group, and the difference was statistically significant. In total, 168 CpG were identified, of which 47 showed up-regulated methylation level and 121 down-regulated. The differences of methylation level between CBP cases and the healthy individuals in the up-regulated gene loci were not statistically significant. The differences of methylation level in some down-regulated gene loci between CBP and the healthy individuals were statistically significant. Among the 168 loci, methylation level in the case group was lower than in the control group in 79 loci, and the differences were statistically significant (all *P* < 0.05). There were significantly fewer mtDNA methylations in patients with CBP than in healthy individuals in the following regions: RNR1, RNR2, TRNQ, TRNM, TRNW, TRNS1, ND2, ND5, CO1, CO2, CO3, ATP6 and CYB (all *P* < 0.05; Figure 1). KEGG pathway analyses identified significantly enriched pathways in the differentially methylated CpG, including aminoacyl–tRNA biosynthesis, oxidative phosphorylation, Parkinson's disease, ribosome biogenesis in eukaryotes, *Staphylococcus aureus* infection, retrograde endocannabinoid signaling, pertussis, ribosome, thermogenesis, complement and coagulation cascades, systemic lupus erythematosus, Alzheimer's disease, Huntington disease, metabolic pathways signaling pathway (Figure 2). The mtDNA methylation levels from the CBP patients [median ± IQR, 25th percentile, 75th percentile: (1.140 ± 0.570, 0.965, 1.535)%] were significantly lower than those from the healthy individuals [median ± IQR, 25th percentile, 75th percentile: (1.705 ± 0.205, 1.240, 2.445)%; *U* = 525, *P* = 0.018] (Figure 3).

**Figure 4:**
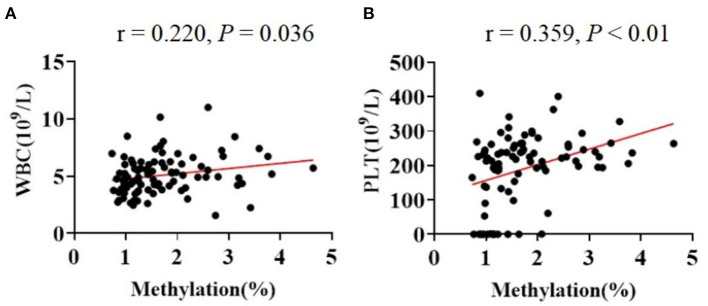
The Spearman correlations among the indicators in all participants. The line in red represented a positive correlation. **(A)** WBC and methylation (*r* = 0.220, *P* = 0.036). **(B)** PLT and methylation (*r* = 0.359, *P* < 0.05).

**Figure 5:**
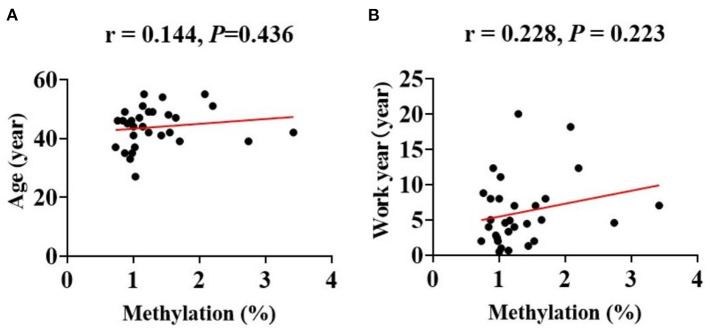
The Spearman correlation analysis among the CBP patients. The line in red represented correlation trend line. **(A)** Age (year) and methylation percentage (*r* = 0.144, *P* = 0.436), **(B)** work year (year) and methylation percentage (*r* = 0.228, *P* = 0.223).

Additionally, the Spearmans correlation coefficient showed that the mtDNA methylation levels were positively correlated with the counts of circulating leukocytes [WBC (*r* = 0.220, *P* = 0.036)] and of platelets [PLT (*r* = 0.359, *P* < 0.01); Figure 4]. The Spearman correlation analysis among all patients. (a) Correlation between age and methylation levels in CBP (*r* = 0.144, *P* = 0.436). (b) Correlation between work year and methylation levels in CBP (*r* = 0.228, *P* = 0.223; Figure 5).

## Discussion

The results of this study revealed that CBP patients had significantly aberrant/lower mtDNA methylation level compared to the healthy individuals. This finding highlights the effect of CBP on mtDNA methylation and peripheral blood cells which may provide clues for further research on the mechanisms of peripheral blood cytopenia during the development of CBP.

Benzene exposure, both occupational and non-occupational, has the potential to cause cancer in humans (18). Early diagnosis and management of CBP have always been a challenge to clinicians. The diagnosis standard of CBP is based on a history of exposure, clinical presentation, and change in blood indices. However, these diagnostic features are difficult to determine in the early stage of the disease. Although molecular diagnostic methods have been explored for use in auxiliary diagnosis and toxicological mechanism studies of CBP, there is still much for improvement. This study performed a comprehensive mtDNA methylation analysis to better identify biomarkers of aberrant DNA methylation in CBP. DNA methylation is a stable epigenetic mark involved in the regulation of gene expression and disease pathophysiology (19). Some studies have suggested that the methylation of specific loci in DNA from body fluid samples has been found useful in cancer diagnosis and prognosis (20). DNA damage and subsequent dysfunction in the mitochondrion are important factors in a range of human diseases due to their roles in cell metabolism (21). Thus, there is increasing interest in the role of mitochondrial epigenetics in common diseases associated with mitochondrial dysfunction (22). In this regard, several studies have searched for relationships between mtDNA methylation levels and diseases. For example, reduced mtDNA copy number and increased methylation levels of specific loci at mtDNA have been shown to increase the risk of macrosomia (23). Additionally, mtDNA hypermethylation has been shown to precede diabetic retinopathy, with mtDNA methylation decreasing after the onset of diabetic retinopathy (24). Therefore, mitochondrial epigenetic markers are crucial in early disease detection and disease prevention.

Our research found that mtDNA methylation levels globally decreased in CBP patients. We also analyzed the differences of each methylation site in mitochondria between the two groups. The difference of methylation level between CBP and the healthy individuals in any of the up-regulated gene loci was not statistically significant, but the differences in some of the down-regulated gene loci between the two groups were statistically significant. Therefore, the down-regulated genes were the focus of this study. The article published by Wang et al. (16) suggested that the methylation levels of two sites in the mitochondrial *CO1* gene region were significantly reduced in benzene poisoned patients. This study also found that the methylation levels of multiple sites in the CO1 gene region were significantly reduced in benzene poisoning patients. However, the specific methylation sites in this study are different from those in Wang's previous study. The region reported in the previous study of Wang et al. was examined using pyrosequencing to detect the short sequence of the gene region, while the current study using a different detection method did not include that methylated gene locus. Previous studies suggest that mtDNA methylation levels may have a regulatory effect on the transcription of target proteins (25). This corresponding regulatory process promotes changes in the expression of downstream target proteins, which lead to the generation of mitochondrial reactive oxygen species (ROS) and apoptosis. In addition, during occupational benzene exposure, oxidative stressors such as ROS may elevate because of benzene and benzene metabolites, which may affect the proliferation of cells at the molecular level in the bone marrow by stimulating various epigenetic modifications, resulting in changes in peripheral blood cells counts (26). This study found that decreased mtDNA methylation levels correlated with lower leukocytes and platelets counts, demonstrating that mtDNA methylation may play a critical role in the pathological process of benzene-induced cytopenia. Pathway analysis identified 14 significant pathways involving Aminoacyl–tRNA biosynthesis, Oxidative phosphorylation. Aminoacyl–tRNA catalyzes the tRNA aminoacylation reaction to generate aminoacyl-tRNA, which ensures the speed and accuracy of protein synthesis. Oxidative phosphorylation is the main step in ATP synthesis. Protein and ATP biosynthesis is a major step in leukocyte and platelet production. This also explains the reduced methylation levels of associated mitochondrial regulatory genes in the benzene poisoning population when leukocytes and platelets are reduced to regulate the elevated levels of their target proteins used to replenish essential substances for cell synthesis. In addition, we performed a correlation analysis of age and work year with mean mitochondrial methylation levels in the CBP patients and found no significant correlation. This also indicates that age and work year do not have an effect on the results of this study. The results of this study suggest that changes in mitochondrial methylation levels occur after benzene exposure acts to produce toxicity in the organism, independent of the benzene exposure time-of-action effect. As aforementioned, DNA methylation is crucial for multiple cellular processes, thus it is understandable that its deregulation has been linked to serve diseases. In blood DNA, aberrant methylation is detected as a molecular early screening tool for cancer and other serve diseases. As a result, methylation signatures have the potential to serve as response markers in CBP. Future studies should explore whether the changes in mtDNA methylation level occur earlier than in peripheral blood cells counts.

The study also has several limitations. First, the sample size of the case group is relatively limited, thus the findings need to be further validated. Second, this study did not investigate the temporal relationship between the duration of benzene exposure and mtDNA methylation; such a relationship would aid in determining the earliest time when mtDNA modifications occur in those who are at risk. Third, the blood sample we used contained different types of cells, which might lead to confounding, and mtDNA alterations in underrepresented cells might be underestimated.

In conclusion, this study found that CBP might be associated with aberrant/lower mtDNA methylation level which was positively correlated with the counts of circulating leukocytes and platelets. The findings may provide clues for further research on the mechanisms of peripheral blood cytopenia during the development of CBP.

## Data availability statement

The datasets presented in this study can be found in online repositories. The names of the repository/repositories and accession number(s) can be found below: [https://www.ncbi.nlm.nih.gov/sra/PRJNA877783].

## Ethics statement

The studies involving human participants were reviewed and approved by the Ethical Committee of Shenzhen Prevention and Treatment Center for Occupational Diseases. The patients/participants provided their written informed consent to participate in this study.

## Author contributions

DW and DL: conceptualization. GF and XY: investigation and data curation. GF: formal analysis and methodology. WZ and NZ: supervision. PL, ZZ, and YW: writing–original draft. DW, DL, and YG: writing–review and editing. All authors contributed to the article and approved the submitted version.

## References

[1] North M Tandon VJ Thomas R Loguinov A Gerlovina I Hubbard AE. Genome-wide functional profiling reveals genes required for tolerance to benzene metabolites in yeast. PLoS ONE. (2011) 6:e24205. 10.1371/journal.pone.0024205 21912624 PMC3166172

[2] Chen L Guo P Zhang H Li W Gao C Huang Z. Benzene-induced mouse hematotoxicity is regulated by a protein phosphatase 2A complex that stimulates transcription of cytochrome P4502E1. J Biol Chem. (2019) 294:2486–99. 10.1074/jbc.RA118.006319 30567741 PMC6378973

[3] Ross D. The role of metabolism and specific metabolites in benzene-induced toxicity: evidence and issues. J Toxicol Environ Health A. (2000) 61:357–72. 10.1080/00984100050166361 11086940

[4] Poca KSD Giardini I Silva PVB Geraldino BR Bellomo A Alves JA. Gasoline-station workers in Brazil: benzene exposure; genotoxic and immunotoxic effects. Mutat Res Genet Toxicol Environ Mutagen. (2021) 865:503322. 10.1016/j.mrgentox.2021.503322 33865537

[5] Singh MP Ram KR Mishra M Shrivastava M Saxena DK Chowdhuri DK. Effects of co-exposure of benzene, toluene and xylene to *Drosophila melanogaster*: alteration in hsp70, hsp60, hsp83, hsp26, ROS generation and oxidative stress markers. Chemosphere. (2010) 79:577–87. 10.1016/j.chemosphere.2010.01.054 20188393

[6] Gaikwad AS Mahmood R Beerappa R Karunamoorthy P Venugopal D. Mitochondrial DNA copy number and cytogenetic damage among fuel filling station attendants. Environ Mol Mutagen. (2020) 61:820–9. 10.1002/em.22404 32816342

[7] Lee HC Yin PH Lu CY Chi CW Wei YH. Increase of mitochondria and mitochondrial DNA in response to oxidative stress in human cells. Biochem J. (2000) 348(Pt 2):425–32. 10.1042/bj3480425 10816438 PMC1221082

[8] Park J Choi C. Contribution of mitochondrial network dynamics to intracellular ROS signaling. Commun Integr Biol. (2012) 5:81–3. 10.4161/cib.18257 22482018 PMC3291322

[9] Liu R Zhang L McHale CM Hammond SK. Paternal smoking and risk of childhood acute lymphoblastic leukemia: systematic review and meta-analysis. J Oncol. (2011) 2011:854584. 10.1155/2011/854584 21765828 PMC3132639

[10] Zheng M Lin F Hou F Li G Zhu C Xu P. Association between promoter methylation of gene ERCC3 and benzene hematotoxicity. Int J Environ Res Public Health. (2017) 14:921. 10.3390/ijerph14080921 28813025 PMC5580623

[11] Jamebozorgi I Majidizadeh T Pouryagoub G Mahjoubi F. Aberrant DNA methylation of two tumor suppressor genes, p14(ARF) and p15(INK4b), after chronic occupational exposure to low level of benzene. Int J Occup Environ Med. (2018) 9:145–51. 10.15171/ijoem.2018.1317 29995020 PMC6466977

[12] Hu J Ma H Zhang W Yu Z Sheng G Fu J. Effects of benzene and its metabolites on global DNA methylation in human normal hepatic L02 cells. Environ Toxicol. (2014) 29:108–16. 10.1002/tox.20777 21953684

[13] Ji Z McHale CM Bersonda J Tung J Smith MT Zhang L. Induction of centrosome amplification by formaldehyde, but not hydroquinone, in human lymphoblastoid TK6 cells. Environ Mol Mutagen. (2015) 56:535–44. 10.1002/em.21947 25821186 PMC6529207

[14] Yang J Bai W Niu P Tian L Gao A. Aberrant hypomethylated STAT3 was identified as a biomarker of chronic benzene poisoning through integrating DNA methylation and mRNA expression data. Exp Mol Pathol. (2014) 96:346–53. 10.1016/j.yexmp.2014.02.013 24613686

[15] Xing C Wang QF Li B Tian H Ni Y Yin S Li G. Methylation and expression analysis of tumor suppressor genes p15 and p16 in benzene poisoning. Chem Biol Interact. (2010) 184:306–9. 10.1016/j.cbi.2009.12.028 20044985

[16] Wang DP Cai DY Yang XL Lu X Lin DF Li PM. [Study of methylation of mitochondrial MT-COI of benzene poisoning]. Zhonghua Lao Dong Wei Sheng Zhi Ye Bing Za Zhi. (2020) 38:664–8. 10.3760/cma.j.cn121094-20200409-00174 33036528

[17] Wang D Yang X Zhang Y Lin D Li P Zhang Z. Platelet mitochondrial cytochrome c oxidase subunit I variants with benzene poisoning. J Thorac Dis. (2018) 10:6811–8. 10.21037/jtd.2018.11.82 30746226 PMC6344673

[18] Mandal S Sen PK Peddada SD. A hierarchical functional data analytic approach for analyzing physiologically based pharmacokinetic models. Environmetrics. (2013) 24:172–9. 10.1002/env.2198 23682216 PMC3652487

[19] Mendizabal I Zeng J Keller TE Yi SV. Body-hypomethylated human genes harbor extensive intragenic transcriptional activity and are prone to cancer-associated dysregulation. Nucleic Acids Res. (2017) 45:4390–400. 10.1093/nar/gkx020 28115635 PMC5416765

[20] Ye M Huang T Ying Y Li J Yang P Ni C. Detection of 14-3-3 sigma (sigma) promoter methylation as a noninvasive biomarker using blood samples for breast cancer diagnosis. Oncotarget. (2017) 8:9230–42. 10.18632/oncotarget.13992 27999208 PMC5354727

[21] Jayabal P Ma C Nepal M Shen Y Che R Turkson J. Involvement of FANCD2 in energy metabolism *via* ATP5alpha. Sci Rep. (2017) 7:4921. 10.1038/s41598-017-05150-1 28687786 PMC5501830

[22] Byun HM Baccarelli AA. Environmental exposure and mitochondrial epigenetics: study design and analytical challenges. Hum Genet. (2014) 133:247–57. 10.1007/s00439-013-1417-x 24402053 PMC4351743

[23] Lin XJ Xu XX Zhang HX Ding MM Cao WQ Yu QY. Placental mtDNA copy number and methylation in association with macrosomia in healthy pregnancy. Placenta. (2022) 118:1–9. 10.1016/j.placenta.2021.12.021 34972066

[24] Huang Y Chi J Wei F Zhou Y Cao Y Wang Y. Mitochondrial DNA: a new predictor of diabetic kidney disease. Int J Endocrinol. (2020) 2020:3650937. 10.1155/2020/3650937 32733553 PMC7378596

[25] Xiao CL Zhu S He M Chen D Zhang Q Chen Y. N-methyladenine DNA modification in the human genome. Mol Cell. (2018) 71:306–18 e7. 10.1016/j.molcel.2018.06.015 30017583

[26] Xue P Gao L Xiao S Zhang G Xiao M Zhang Q. Genetic polymorphisms in XRCC1, CD3EAP, PPP1R13L, XPB, XPC, and XPF and the risk of chronic benzene poisoning in a Chinese occupational population. PLoS ONE. (2015) 10:e0144458. 10.1371/journal.pone.0144458 26681190 PMC4683048

