# Supplementary material for: Impact of chronic benzene poisoning on aberrant mitochondrial DNA methylation: A prospective observational study
Source: Front Public Health. 2023 Feb 3;11:990051. doi: 10.3389/fpubh.2023.990051 (PMC9937586; doi:10.3389/fpubh.2023.990051)
Supplement: Supplementary file 1 [file Table_1.DOCX]

**Supplementary Table S1** Primer sequences

| targetSeq | ForwardPrimer | Start | End | ReversePrimer | Start | End |
| --- | --- | --- | --- | --- | --- | --- |
| chrM | TTTTTTATGTATTTGGTATTTTYGTTTGGGG | 39 | 69 | ATATCCTACAAACATTAATTAATTAACACACTTTA | 199 | 233 |
| chrM | GTTTAGAYGGGTTTATATTATTTTATAAATAAATAGG | 619 | 655 | CAACCCTAAAATTAATATAACTTAATTAAACTTTC | 845 | 879 |
| chrM | GYGTAAAGAGTGTTTTAGATTATTTTTTTTTTAATAAAG | 936 | 974 | CATAATAAAATATCTAATCCCAATTTAAATCTTAAC | 1059 | 1094 |
| chrM | GTTTAAAATTTAAAGGATTTGGYGGTGTTTT | 1157 | 1187 | CTTTATAACCTTCATCAAAATTTACTAAAAATAAC | 1265 | 1299 |
| chrM | AAGAYGTTAGGTTAAGGTGTAGTTTATG | 1320 | 1347 | CTCTACTCTCAATTTACTACTAAATCC | 1423 | 1449 |
| chrM | TATGGTAAGTGTATTGGAAAGTGTATTTG | 1568 | 1596 | CTATATTATACTTAATTATAATTTTTCATCTTTCCC | 1793 | 1828 |
| chrM | TTTTGTAAGGAGAGTTAAAGTTAAGATTTTYGAAATTAG | 1877 | 1915 | CTTAAACAACCAACTATCACCAAACT | 2005 | 2030 |
| chrM | AGGAATAGTTTTTTGGATATTAGGAAAAAATTTTG | 2108 | 2142 | TCAATTCAATATTTTAATCTAACRCAAACTTATAC | 2335 | 2369 |
| chrM | ATGTTTATAAGGAAAGGTTAAAAAAAGTAAAAGG | 2440 | 2473 | CTATTTAAAAAACAAATAATTATACTACCTTTACAC | 2587 | 2622 |
| chrM | TTTTAYGAGGGTTTAGTTGTTTTTTATTTTTAATTAG | 2639 | 2675 | CATATACTACTCRAAAATTAAATTCTACTCC | 2827 | 2857 |
| chrM | ATAAGTTATTTTAGGGATAATAGYGTAATTTTATTTTAG | 2921 | 2959 | CTTTATAAAATAAACCTTATTTCTCTTATCCTTTC | 3128 | 3162 |
| chrM | GGGTTTGTTAAGATGGTAGAGTTYGGTAATYGTATAAAA | 3225 | 3263 | CCTAAAATTTTTCRTTCRATAAACATTAAAAATACC | 3358 | 3393 |
| chrM | TAAYGTTGTAGGTTTTTAYGGGTTATTATAATTTT | 3418 | 3452 | CCCTAATCAAAAAATTAAATAAACRACTAAACTAA | 3633 | 3667 |
| chrM | TTTTTAGGATTATGAGAATYGAATTTATTTTTGAG | 4327 | 4361 | CATATTTATTTCTAAACCTACTCAAATAAAAAATC | 4547 | 4581 |
| chrM | TTTTTGAGTTTTAGAGGTTATTTAAGGTATTTTTTTG | 4806 | 4842 | CTAAATTTAATTTAATCCACCTCAACTAC | 4964 | 4992 |
| chrM | TTAGTAAGTTGTAATATTTAATTTTTGTAATAGTTAAGG | 5554 | 5592 | ACTTCAAACCTACCRAAACTTCTCCC | 5757 | 5782 |
| chrM | ATTTYGGAGTTGGTAAAAAGAGGTTTAATTTTTG | 5817 | 5850 | ACTATACCTAAAACTCCAACTCATAC | 5974 | 5999 |
| chrM | GAGTTGGGTTAGTTAGGTAATTTTTTAG | 6022 | 6049 | CRATTATTAAAAAAACTAATCAATTACCAAAACCT | 6132 | 6166 |
| chrM | GTYGGAGTAGGAATAGGTTGAATAGT | 6262 | 6287 | AAAATTAATAACCCCTAAAATAAAAAAAACACCTA | 6362 | 6396 |
| chrM | GAGGAGGAGATTTTATTTTATATTAATATTTATTTTG | 6575 | 6611 | CATACCTATATATCCAAATAATTCTTTTTTTCC | 6691 | 6723 |
| chrM | TATAGTAGGAATAGAYGTAGATATAYGAGTATAT | 6783 | 6816 | CAAAACACTACAACAAATCATTTCATATTAC | 6893 | 6923 |
| chrM | GTAGGTGGTTTGATTGGTATTGTATT | 6952 | 6977 | CCTCCTATAATAACAAATACAACTCC | 7054 | 7079 |
| chrM | GTTTTAATAGTAGAAGAATTTTTTATAAATTTGGAG | 7348 | 7383 | CCATAAAATTAACTTAAAACCAACTTTAAAAAATTC | 7464 | 7499 |
| chrM | TATGTAGYGTAAGTAGGTTTATAAGAYGTTATTTT | 7593 | 7627 | AACAAAATAATTCAAACRATTTCTATTTCCTAAAC | 7758 | 7792 |
| chrM | TTTTATTATTTTTAGAATTAGGYGATTTGYGATTTTTTG | 7957 | 7995 | CATCTATTTTTAAACCTAATATAAAAACAACTCAT | 8072 | 8106 |
| chrM | GAGYGGGYGTAGTGATTATAGGTTTT | 8853 | 8878 | CTTCCAATTAAATACATAAATAAATAACCTACAAT | 9020 | 9054 |
| chrM | TGATTTTTAATAGGGGTTTTTTTAGTTTTTTTAATG | 9253 | 9288 | CTAAAATAATAAATAAAATTATCCCRTATCRAAAACCTT | 9437 | 9475 |
| chrM | TATTATTTTAGTTTAGTTTTTATTTTTTAATTAGGAGGG | 9511 | 9549 | CTATTAAACTATAATAAACTCAAATAATTAATACTCC | 9628 | 9664 |
| chrM | TTTTGTAGTTATAGGTTTTTAYGGATTTTAYGTTATTAT | 9798 | 9836 | CCACATCTACAAAATACCAATATCAAAC | 9922 | 9949 |
| chrM | TATTTATTGATGAGGGTTTTATTTTTTTAGTATAAATAG | 9972 | 10010 | CCRCACTCRTAAAAAATAAATTTTTCTATATAACC | 10144 | 10178 |
| chrM | TTTGGTTTATGAGTGATTATAAAAAGGATTAG | 10362 | 10393 | CTATAATAAACAACRATAATATTATTCCTTCTAAAC | 10565 | 10600 |
| chrM | TGAGGTAATTAGTTAGAAYGTTTGAAYGTAGGTAT | 11166 | 11200 | CTTCRACATAAACTTTAAAAAATCATAAATAAAATCC | 11391 | 11427 |
| chrM | AATAGTTATTTATTGGTTTTAGGTTTTAAAAATTTTGG | 12280 | 12317 | CAATTCRAAATAATAACTTCTTAATCTAAACACAT | 12500 | 12534 |
| chrM | GTTTATYGGTTGAGAGGGYGTAGGAAT | 12757 | 12783 | CTACTACTACTAAAAAAAAACCTAATAATAAAATAAAAC | 12963 | 13001 |
| chrM | TTTAGTTTTATTTTATTTAAGTATTATAGTTGTAGTAGG | 13066 | 13104 | AAAACRCAAACTACTACRAACAAAATAATAATAAC | 13181 | 13215 |
| chrM | GTTTAGTATTAGTAGGAATATTTTTTTTTATAGGT | 13467 | 13501 | CTTCCRACTACCAAACRTTTAATAAAATTTAATAA | 13685 | 13719 |
| chrM | TYGGTTTATTTTTTGGYGTTTGTTTGATTTTTTAA | 14845 | 14879 | CCATTAACRTAAAAATAACRAATAATTCAACCATA | 14970 | 15004 |
| chrM | TATAGGTTATGTTTTTTYGTGAGGTTAAATATTATTTTG | 15131 | 15169 | TCRTATAAAAATAAAACTATCTACTAAATAACCTC | 15244 | 15278 |
| chrM | TTTGAATYGGAGGATAATTAGTAAGTTATTTTTTT | 15754 | 15788 | CTACAAAAACAAACCCATTTAAATATTTTATTTTC | 15864 | 15898 |
| chrM | TTAATTTAAATTATTTTTTGTTTTTTTATGGGGAAGTAG | 16005 | 16043 | CTACAAATAATCAAATATTTATAATACCRTACAATATTC | 16119 | 16157 |
| chrM | TTAGATAGGGGTTTTTTGATTATTATTTTTYGTGAAATT | 16383 | 16421 | TAAACTTTATAACCCTAAAATAAAAACCAAATATC | 16498 | 16532 |

chrM :Chromosome Mitochondrial
